# Time to adjuvant chemotherapy and overall survival in advanced-stage ovarian cancer patients in England: a population-based retrospective cohort study

**DOI:** 10.1016/j.esmorw.2025.100143

**Published:** 2025-04-28

**Authors:** L. Steventon, S. Nicum, K. Man, D. Dodwell, Z. Wang, A. Patel, B. Pickwell-Smith, L. Wei, P. Chambers

**Affiliations:** 1 Research Department of Practice and Policy, UCL School of Pharmacy, London, UK; 2 University College London Hospital NHS Foundation Trust, London, UK; 3 Nuffield Department of Population Health, University of Oxford, Oxford, UK; 4 Leeds Teaching Hospitals NHS Trust, Leeds, UK

**Keywords:** cancer, ovarian, surgery, adjuvant chemotherapy, treatment equity

## Abstract

**Background and purpose:**

In advanced ovarian cancer, delayed time to chemotherapy (TTC) has been associated with poorer survival outcomes; evidence is conflicting, however. This study investigated the impact of patient demographic factors on TTC and assessed whether TTC was associated with 5-year overall survival.

**Materials and methods:**

A retrospective cohort study was conducted using English national data for women with advanced-stage ovarian cancer (IIB-IV), treated with primary debulking surgery (PDS) or interval debulking surgery (IDS) + adjuvant carboplatin/paclitaxel chemotherapy between 1 January 2014 and 31 December 2019. Cox proportional hazards regression was used to compare the primary outcome of 5-year overall survival between patients treated within ≤6 weeks (0-42 days) or >6 weeks (>42 days) of surgery.

**Results:**

A total of 4619 patients were included. Of these, 42% (*n* = 1940) received PDS and 58% (*n* = 2679) IDS. Median TTC was 45 days [interquartile range (IQR) 37-55 days] for PDS and 34 days (IQR 27-42 days) for IDS. TTC ≤6 weeks was associated with 5-year survival in the IDS cohort (HR 1.18, 95% CI 1.06-1.33, *P* = 0.003), but not in the PDS cohort (HR 1.04, 95% CI 0.90-1.21, *P* = 0.6). A higher proportion of patients from the most socioeconomically deprived backgrounds waited >6 weeks (45%, *n* = 291) compared with the least deprived (37%, *n* = 398). Adjuvant chemotherapy was initiated in 72% of patients in London within ≤6 weeks compared with 47% in the North West.

**Conclusions:**

Median TTC exceeded 4-week guidance from the European Society of Medical Oncology. TTC >6 weeks was associated with reduced 5-year survival in patients treated with interval surgery, but not primary surgery. Regional disparities in TTC were observed.

## Highlights

• TTC after surgery may affect survival outcomes.
• TTC was investigated in advanced ovarian cancer using English national data.
• Proportionally, more women in London were treated in ≤6 weeks than other regions.
• TTC >6 weeks from interval surgery impacted survival (HR 1.18, 95% CI 1.06-1.33).
• TTC did not impact survival after primary surgery (HR 1.04, 95% CI 0.9-1.21).

## Introduction

Cytoreductive (debulking) surgery and chemotherapy are the mainstay of treatment of ovarian cancer.^1^ Complete cytoreduction—the resection of all visible macroscopic disease—has been demonstrated to lead to improved survival,^2^ with a meta-analysis of clinical trials demonstrating that the aim of surgery should always be complete resection, to achieve optimal outcomes.^3^ Surgical resection may be carried out upfront [primary debulking surgery: (PDS)], or following neoadjuvant chemotherapy [interval debulking surgery: (IDS)]. Neoadjuvant ± adjuvant carboplatin/paclitaxel chemotherapy is recommended for all patients with stage II-IV ovarian cancer, excluding patients ineligible for surgery due to widespread disease and low likelihood of complete resection, where single-modality chemotherapy may be offered.^4^ Maintenance therapies with bevacizumab and poly (ADP-ribose) polymerase inhibitors (PARPi) are now given following chemotherapy in first-line and recurrent settings, after significant improvements in progression-free survival were demonstrated in the ICON7,^5^ PAOLA-1^6^ and other pivotal clinical trials.

Several studies have investigated the time period from surgical resection to initiation of adjuvant chemotherapy, termed time to chemotherapy (TTC), assessing whether prolonged TTC reduces survival time. In advanced-stage epithelial ovarian cancer [defined as International Federation of Gynecology and Obstetrics (FIGO) 2014 stages IIB-IV^7^] according to the commonly used definition of advanced disease in published randomised controlled trials (RCTs),^8^ TTC >37 days was associated with poorer overall survival in patients with no residual disease in both primary and interval debulking cohorts.^9^ A large retrospective analysis reported an optimal period of 20-39 days to initiate adjuvant treatment following primary surgery (42.9% surviving for 5 years) and observing poorer survival when TTC exceeded >40 days (38.2%).^10^ Conversely, an analysis of clinical trials in early-stage ovarian cancer (FIGO stages I-II) by the Gynaecologic Oncology Group reported no association between TTC and overall survival, suggesting adjuvant chemotherapy timing was not a significant prognostic factor in the early-stage, primary surgery setting [hazard ratio (HR) 0.90, 95% CI 0.59-1.37 for TTC 2-4 weeks and HR 0.72, 95% CI 0.46-1.13 for TTC >4 weeks].^11^ Rocher et al. also demonstrated no association between a TTC period of <6 or >6 weeks with relapse-free or overall survival in both early- and advanced-stage disease (HR 0.99, 95% CI 0.91-1.09)^12^ These findings suggest the relationship between TTC and survival outcomes in ovarian cancer may be more significant in advanced-stage than early-stage disease.

Given the potential impact of the TTC period, it is important to consider patient factors that may impact the time taken for a patient to initiate adjuvant treatment. Disparity in engagement with health care is well documented relative to demographic factors such as ethnicity, age, socioeconomic status and geographical region, with observed differences in time to receive a diagnosis,^13^ knowledge and identification of symptoms,^10^ and reporting of symptoms to clinicians^14^^,^^15^ relative to demography. Even within the socialised UK National Health Service (NHS), patients from deprived areas have been found to be less likely to undergo chemotherapy or surgery for ovarian cancer.^16^ Given findings of significant associations of prolonged TTC with survival outcomes in some studies, it is important to understand if there are delays to starting adjuvant chemotherapy in England and whether there are disparities in adjuvant chemotherapy timing within demographic subgroups of the English ovarian cancer population.^16^ This represents a knowledge gap addressed by this study using national observational data.

## Materials and methods

### Data sources

National, population-level registries for advanced-stage ovarian cancer patients treated within the NHS of England were used. The National Disease Registration Dataset (NCRD) records all cancer diagnoses in England, containing tumour-specific diagnostic information and patient characteristics such as age, ethnicity and socioeconomic status.^17^ Death dates are provided by the Office for National Statistics.^18^ The Systemic Anti-Cancer Therapy Dataset (SACT) records systemic therapy treatments, including drug given, treatment date and dose. Hospital Episode Statistics (HES) datasets record outpatient appointments and hospital admissions, and were used to identify dates of debulking surgery.^19^ Data resource profiles have been published for all datasets, providing detailed descriptions of data quality and near total population-level coverage.^17^^,^^19^^,^^20^ These sources have been appropriately used for other research of this kind.^21^^,^^22^

### Study design and period

This was a national, population-based study using a retrospective cohort study design. The study period of 1 January 2014 to 31 December 2019 was chosen to cover the period where SACT data reporting became mandated and to allow for a sufficiently long follow-up period to capture 5-year overall survival outcomes for patients treated within this period.

### Inclusion and exclusion criteria

Female patients aged ≥18 years with a newly diagnosed primary diagnosis of ovarian, fallopian tube or primary peritoneal cancer of gynaecological origin, specified by ICD-10 codes C56, C57 or C48 in the NCRD, were eligible. Patients with morphology codes specifying sarcoma or borderline tumours were excluded. All histologies were included and patients diagnosed with a previous malignancy were eligible. Tumour grade data were not available in the data sources and were therefore not used to define inclusion criteria.

Patients receiving standard first-line treatment of either PDS and adjuvant carboplatin and paclitaxel [Taxol; Accord UK Ltd (Barnstaple, UK), Fresensius Kabi Ltd (Bad Homburg, Germany), Pfizer Ltd (Tadworth, UK), Seacross Pharmaceuticals Ltd (Stanmore, UK)] doublet chemotherapy, or IDS and neoadjuvant chemotherapy for advanced ovarian cancer (FIGO stages IIB-IV) in the period 1 April 2014 to 31 December 2019 were included. Adjuvant SACT is not necessarily recommended for some subgroups of patients diagnosed with FIGO stages 1A-IC^23^^,^^24^ and therefore these patients were excluded.

To correctly identify first-line treatments, only patients initiating chemotherapy within 90 days of ovarian cancer diagnosis were included. Patients must also have initiated adjuvant chemotherapy within 90 days of PDS or IDS were eligible, as this is the expected period for initiation of adjuvant chemotherapy based on oncologists’ experience of clinical practice in England, and ensures that surgery and chemotherapy treatments are part of the same treatment course. Chemotherapy initiated >6 months after surgery was considered part of a recurrent treatment regimen and, therefore, not adjuvant treatment in the first-line setting, and these patients were excluded as per similar published research.^23^^,^^25^ Patients may have received bevacizumab and/or PARPi maintenance therapy following first-line chemotherapy.

Data cleaning and linkage procedures and specification of the study population are described in the Supplementary Materials (available at https://doi.org/10.1016/j.esmorw.2025.100143), with descriptions of key variables given.

### Study exposures and outcome

The study exposure was time from surgery to initiation of adjuvant carboplatin and paclitaxel doublet chemotherapy: time to chemotherapy (TTC). The primary study outcome was 5-year overall survival defined as death by any cause. The 6-week threshold value was based on the exploration of the dataset to identify median TTC in the dataset, to provide a sufficient sample size to compare 5-year survival between the two groups, and other literature using 37 days,^9^ 40 days^10^ and 6 weeks^12^ as the threshold. Disease-free/progression-free survival were not investigated as the available data sources do not capture these data.

### Statistical analysis plan

The study population was stratified into PDS or IDS cohorts. Median time in days from surgery to initiation of adjuvant carboplatin and paclitaxel doublet chemotherapy was calculated for all patients and described by ethnic group, age category, index of multiple deprivation and region in subgroup analyses.

Patients were followed up for 5 years from date of first adjuvant chemotherapy until the primary study outcome of death by any cause. Patients were censored at their last known follow-up date if they survived for the 5-year follow-up period. Kaplan–Meier survival estimates were used to compare 5-year overall survival between demographic groups, stratified by initiation of adjuvant chemotherapy up to 6 weeks (≤6 weeks, 0-42 days inclusive) or after 6 weeks (>6 weeks, >42 days) from date of surgery.

Cox proportional hazards regression analysis was used to calculate proportional risks of TTC >6 weeks on 5-year overall survival, adjusting for confounding variables of age at time of diagnosis, body mass index (BMI), FIGO cancer stage (IIB-IV), Charlson comorbidity index,^26^ ethnicity (according to UK census groupings, or labelled as ‘Unknown’ if not recorded), NHS commissioning region (as previously described), index of multiple deprivation (IMD) as a weighted score of comorbidity impacting on mortality, and treating centre type (academic or local hospital) derived from NHS organisation code linked to SACT treatment. Bevacizumab maintenance therapy was incorporated into Cox regression analysis as an explanatory covariate. Further details of statistical tests and missing data handling are given in the Supplementary Materials (available at https://doi.org/10.1016/j.esmorw.2025.100143).

### Complete-case analysis

Complete-case analyses were carried out for patients with recorded Eastern Cooperative Oncology Group (ECOG) performance status (PS), to compare with the primary Cox regression analysis and provide an assessment of the impact of TTC both in the presence and absence of recorded PS, which was highlighted as an important prognostic factor in the study planning phase.

### Missing data handling

The extent of missing data for key variables and methodology to handle missingness are described in the Supplementary Materials (available at https://doi.org/10.1016/j.esmorw.2025.100143).

### Patient and public involvement

Patient representation was considered in the planning of this study. A patient representative was consulted on the design of this study and selection of time period, based on their experience of treatment pathways in the NHS. Results of the first analysis were discussed with this patient representative and feedback was considered in the drafting of this manuscript. Findings will be discussed with UK ovarian cancer charity Ovacome, pending successful publication in a peer-reviewed journal.

### Ethics approval and consent to participate

Ethical approval for use of NCRD, SACT and HES data for research into risks and benefits of cancer treatment was given favourable opinion on 10 June 2019, REC reference 19/NS/0057.

## Consent for publication

No individual data is contained within this manuscript and publication is allowed under the terms of agreement.

## Results

**Figure 1:**
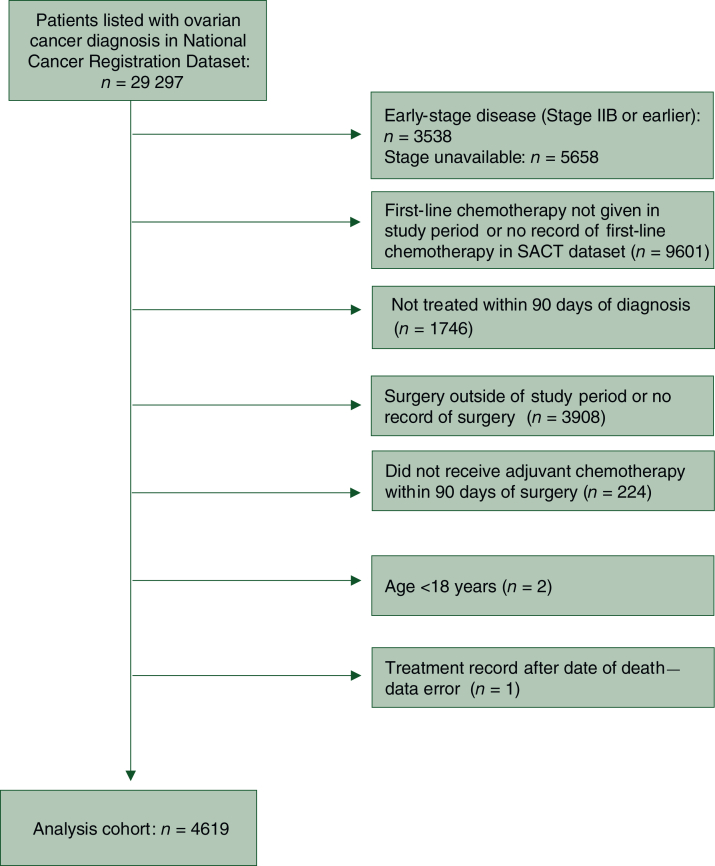
Flowchart showing exclusion criteria to define the study cohort.

A total of 4619 patients were included in the study. Exclusion criteria are shown as a flowchart (Figure 1). Median age at diagnosis was 64 years [interquartile range (IQR) 56-70 years]. Of the patients, 58% received interval surgery (*n* = 2679) and 42% primary surgery (*n* = 1940). Bevacizumab maintenance therapy was received by 32% of patients (*n* = 1489) following first-line carboplatin/paclitaxel. Median TTC was 45 days (IQR 37-55 days) for patients undergoing PDS and 34 days for IDS (IQR 27-42 days). Adjuvant chemotherapy within ≤6 weeks of surgery was received by 61% of patients (*n* = 2824), with TTC >6 weeks for 39% (*n* = 1795). Median follow-up time for the whole study cohort was 2.92 years (IQR 1.95-4.09 years) and was comparable between patients with TTC ≤6 weeks (2.94 years, IQR 1.99-4.11 years) and TTC >6 weeks (2.88 years, IQR 1.90-4.03 years). Baseline characteristics of the study cohort are given in Table 1, stratified by surgical modality setting.

**Table 1:** Baseline characteristics of the study cohort stratified by surgical modality

Characteristic	Overall *N* = 4619^a^	Interval surgery *n* = 2679^a^	Primary surgery *n* = 1940^a^	*P* value^b^
Age, [years, interquartile range (IQR)]	64 (56-70)	65 (58-71)	62 (53-69)	<0.001
<60, *n* (%)	1647 (36)	794 (30)	853 (44)	
60-70, *n* (%)	1659 (36)	1047 (39)	612 (32)	
>70, *n* (%)	1313 (28)	838 (31)	475 (24)	
Cancer stage, *n* (%)				<0.001
2	348 (7.5)	12 (0.4)	336 (17)	
3	3123 (68)	1716 (64)	1407 (73)	
4	1148 (25)	951 (35)	197 (10)	
Time to chemotherapy (days, IQR)	38 (30-49)	34 (27-42)	45 (37-55)	<0.001
0-28 days, *n* (%)	959 (21)	868 (32)	91 (4.7)	
29-42 days, *n* (%)	1865 (40)	1156 (43)	709 (37)	
43-56 days, *n* (%)	1180 (26)	458 (17)	722 (37)	
>57 days, *n* (%)	615 (13)	197 (7.4)	418 (22)	
Bevacizumab maintenance, *n* (%)	1489 (32)	1120 (42)	369 (19)	<0.001
Performance status, *n* (%)				0.020
0	1329 (38)	742 (36)	587 (40)	
1	1931 (55)	1167 (56)	764 (52)	
≥2	278 (7.9)	171 (8.2)	107 (7.3)	
Missing, *n*	1081	599	482	
Body mass index (median, IQR)	25.9 (22.9-30.0)	26.0 (22.9-30.1)	25.8 (22.8-29.8)	0.4
Underweight (<18.5), *n* (%)	128 (2.8)	72 (2.7)	56 (2.9)	
Normal weight (18.5-25), *n* (%)	1884 (41)	1085 (41)	799 (41)	
Overweight (25-30), *n* (%)	1451 (31)	840 (31)	611 (31)	
Obese (30-40), *n* (%)	1001 (22)	598 (22)	403 (21)	
Morbidly obese (>40), *n* (%)	155 (3.4)	84 (3.1)	71 (3.7)	
Ethnicity, *n* (%)				0.002
Asian	185 (4.0)	90 (3.4)	95 (4.9)	
Black	62 (1.3)	36 (1.3)	26 (1.3)	
Mixed race	13 (0.3)	4 (0.1)	9 (0.5)	
Other	65 (1.4)	34 (1.3)	31 (1.6)	
Unknown	119 (2.6)	56 (2.1)	63 (3.2)	
White	4175 (90)	2459 (92)	1716 (88)	
Index of multiple deprivation, *n* (%)				<0.001
1—most deprived	649 (14)	335 (13)	314 (16)	
2	825 (18)	465 (17)	360 (19)	
3	992 (21)	569 (21)	423 (22)	
4	1074 (23)	656 (24)	418 (22)	
5—least deprived	1079 (23)	654 (24)	425 (22)	
Region, *n* (%)				<0.001
East of England	658 (14)	410 (15)	248 (1%)	
London	570 (12)	322 (12)	248 (13)	
Midlands	575 (12)	306 (11)	269 (14)	
North East and Yorkshire	913 (20)	480 (18)	433 (22)	
North West	631 (14)	365 (14)	266 (14)	
South East	678 (15)	423 (16)	255 (13)	
South West	594 (13)	373 (14)	221 (11)	
Centre type, *n* (%)				0.017
Academic	3139 (68)	1858 (69)	1281 (66)	
General hospital	1480 (32)	821 (31)	659 (34)	

a n (%), median (IQR).

b Pearson's Chi-squared test; Wilcoxon rank sum test.

Median TTC was greatest in the North West (43 days, IQR 35-55 days) and shortest in London (35 days, IQR 28-44 days). The proportion of patients treated within ≤6 weeks of surgery was greatest in London (72%) compared with 47% in the North West (*P* < 0.001) and 52% in the Midlands (*P* < 0.001). TTC increased with indices of deprivation, with TTC of 41 days (IQR 33-50 days) in the most deprived subgroup compared with 37 days (IQR 29-47 days) in the least deprived; however, this was not a statistically significant difference, with significant overlap of TTC values. TTC is described by all patient demographic factors in Supplementary Table S1, available at https://doi.org/10.1016/j.esmorw.2025.100143. Boxplots comparing TTC by demographic factors are given in Supplementary Figures S1 to S4, available at https://doi.org/10.1016/j.esmorw.2025.100143. Details of missing data handling are given in the Supplementary Materials available at https://doi.org/10.1016/j.esmorw.2025.100143.

### Survival analysis

**Figure 2:**
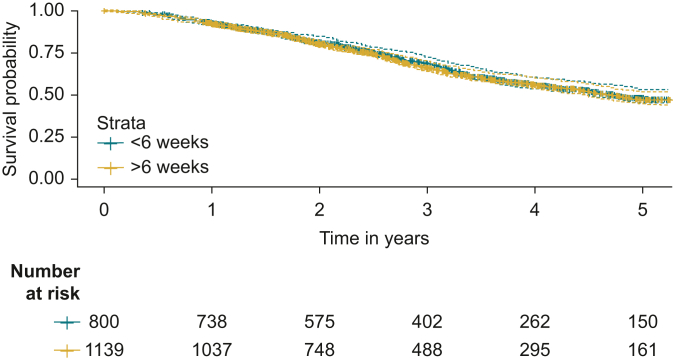
Kaplan–Meier curve comparing 5-year survival in advanced-state ovarian cancer patients treated with primary surgery and adjuvant chemotherapy within ≤6 or >6 weeks.

**Figure 3:**
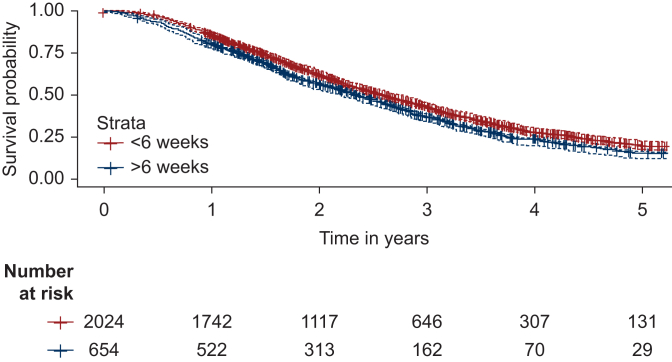
Kaplan–Meier curve comparing 5-year survival in advanced-state ovarian cancer patients treated with interval surgery and adjuvant chemotherapy within ≤6 or >6 weeks.

A total of 4617 patients with recorded follow-up status were included in 5-year overall survival analysis, stratified by surgical modality. Median survival in the PDS cohort was 2.80 years (95% CI 1.73-4.24 years). Median 5-year overall survival was 2.13 years (95% CI 1.29-3.2 years) in the IDS cohort. Kaplan–Meier curves for 5-year overall survival, stratified by surgery type and TTC period, are shown in Figure 2, Figure 3.

Cox proportional hazards regression analysis was carried out for PDS and IDS cohorts in separate analyses, accounting for characteristic differences between these patient groups. Relative to the reference group (treated within ≤6 weeks), the HR was 1.04 in the PDS cohort (95% CI 0.9-1.21, *P* = 0.6) and 1.18 (95% CI 1.06-1.33, *P* = 0.003) in the IDS cohort. In the complete-case analysis including only patients with recorded PS, the HR was 0.99 (95% CI 0.83-1.18, *P* > 0.9) in the PDS cohort and 1.19 (95% CI 1.05-1.36, *P* = 0.008) in the IDS cohort. Covariates associated with 5-year overall survival were age >70 years (HR 1.47, 95% CI 1.122-1.76, *P* < 0.001) in the PDS cohort and age 60-70 years (HR 1.20, 95% CI 1.06-1.35, *P* = 0.003) and >70 years (HR 1.35, 95% CI 1.19-1.53, *P* < 0.001) in the IDS cohort. PS ≤2 and increasing BMI were positively associated with 5-year overall survival in both PDS and IDS cohorts. Results of Cox regression are given for PDS (Table 2) and IDS cohorts (Table 3), including complete-cases analyses including only patients with a recorded PS, derived from clinical notes recording a patient’s ECOG score, assigned at the start of the SACT treatment regimen. In the validation analysis (including only patients with a recorded PS, but excluding PS as an explanatory variable), HR was 0.99 (95% CI 0.83-1.18) for the PDS cohort and 1.19 (95% CI 1.05-1.36) for IDS.

**Table 2:** Cox regression analysis comparing the impact of initiating adjuvant chemotherapy within 6 weeks of primary debulking surgery

	Study cohort (*N* = 1846)			Complete-case analysis (*N* = 1485)		
Primary surgery cohort (*N* = 1939)	HR	95% CI	*P* value^a^	HR	95% CI	*P* value^a^
Time to chemotherapy, weeks						
≤6 (ref)	—	—	—	—	—	
>6	1.04	0.90-1.21	0.6	0.99	0.83-1.18	>0.9
Cancer stage						
IIB (ref)	—	—	—	—	—	
III	2.04	1.59-2.61	<0.001∗∗	1.83	1.36-2.45	<0.001∗∗
IV	2.93	2.12-4.03	<0.001∗∗	2.72	1.86-3.99	<0.001∗∗
Performance status						
0 (ref)	—	—	—	—	—	
1	—	—	—	1.34	1.10-1.62	0.003∗
≥2	—	—	—	2.84	2.14-3.78	<0.001∗∗
Age, years						
<60 (ref)	—	—		—	—	
60-70	1.13	0.95-1.34	0.2	1.13	0.92-1.39	0.2
>70	1.47	1.22-1.76	<0.001∗∗	1.40	1.13-1.74	0.002∗
Body mass index						
Underweight (<18.5)	1.55	1.07-2.25	0.02∗	1.37	0.87-2.15	0.2
Normal weight (18.5-25, ref)	—	—	—	—	—	—
Overweight (25-30)	0.80	0.67-0.95	0.013∗	0.83	0.68-1.02	0.079
Obese (30-40)	0.78	0.64-0.95	0.012∗	0.82	0.65-1.04	0.10
Morbidly obese	0.84	0.53-1.32	0.4	0.97	0.59-1.61	>0.9
Bevacizumab maintenance	0.74	0.62-0.89	0.001∗∗	0.83	0.68-1.02	0.079
Region						
East of England (ref)	—	—	—	—	—	—
London	0.90	0.65-1.23	0.5	1.01	0.69-1.47	>0.9
Midlands	1.07	0.79-1.47	0.7	1.01	0.70-1.47	>0.9
North East and Yorkshire	1.13	0.85-1.50	0.4	1.04	0.74-1.47	0.8
North West	1.18	0.87-1.60	0.3	1.17	0.82-1.67	0.4
South East	1.32	0.96-1.82	0.089	1.19	0.81-1.76	0.4
South West	1.21	0.87-1.68	0.3	1.14	0.76-1.70	0.5
Hospital type						
Teaching hospital (ref)	—	—	—	—	—	
General hospital	1.09	0.91-1.30	0.4	1.15	0.93-1.43	0.2
Ethnicity						
White	—	—	—	—	—	—
Nonwhite		1.00	0.78-1.29	1.02	0.76-1.37	0.9
Index of multiple deprivation						
1—most deprived (ref)	—	—	—	—	—	
2	0.88	0.69-1.12	0.3	0.97	0.74-1.29	0.9
3	0.70	0.55-0.90	0.004	0.69	0.52-0.91	0.009
4	0.77	0.60-0.97	0.028	0.78	0.59-1.03	0.075
5—least deprived	0.71	0.56-0.92	0.008	0.72	0.53-0.96	0.026

Primary and complete-case analysis (including only patients with a recorded performance status) are compared.

CI, confidence interval; HR, hazard ratio.

*∗P* value significant at level of <0.01.

*∗∗P* value significant at level of <0.001.

a Fisher's exact test; Wilcoxon rank sum test; Pearson's Chi-squared test.

**Table 3:** Cox regression analysis comparing the impact of initiating adjuvant chemotherapy within 6 weeks of interval debulking surgery

	Study cohort (*N* = 2678)			Complete-case analysis (*N* = 2080)		
Interval surgery cohort (*N* = 1939)	HR	95% CI	*P* value^a^	HR	95% CI	*P* value^a^
Time to chemotherapy, weeks						
≤6 (ref)	—	—	—	—	—	
>6	1.18	1.06-1.33	0.003	1.19	1.05-1.36	0.008
Cancer stage						
IIB (ref)	—	—		—	—	
III	1.87	0.83-4.18	0.13	1.87	0.77-4.53	0.2
IV	2.06	0.92-4.63	0.081	2.04	0.83-4.97	0.12
Performance status						
0 (ref)	—	—	—	—	—	
1	—	—	—	1.06	0.94-1.20	0.3
≥2	—	—	—	1.64	1.35-2.00	<0.001
Age, years						
<60 (ref)	—	—		—	—	
60-70	1.20	1.06-1.35	0.003	1.23	1.07-1.41	0.004
>70	1.35	1.19-1.53	<0.001	1.36	1.17-1.57	<0.001
Body mass index						
Underweight (<18.5)	1.22	0.93-1.60	0.2	1.28	0.95-1.74	0.11
Normal weight (18.5-25, ref)	—	—	—	—	—	—
Overweight (25-30)	0.90	0.80-1.01	0.064	0.91	0.80-1.04	0.2
Obese (30-40)	0.76	0.67-0.87	<0.001	0.74	0.64-0.86	<0.001
Morbidly obese	0.71	0.54-0.95	0.020	0.72	0.51-1.02	0.061
Bevacizumab maintenance	0.89	0.80-0.98	0.023	0.88	0.73-1.00	0.055
Region						
East of England (ref)	—	—	—	—	—	
London	0.74	0.61-0.89	0.002	0.72	0.58-0.91	0.005
Midlands	0.99	0.82-1.20	>0.9	0.93	0.75-1.16	0.5
North East and Yorkshire	1.04	0.88-1.24	0.6	1.08	0.89-1.31	0.4
North West	0.89	0.74-1.06	0.2	0.91	0.74-1.12	0.4
South East	0.80	0.66-0.96	0.016	0.82	0.66-1.02	0.071
South West	0.91	0.76-1.10	0.3	0.92	0.74-1.15	0.5
Hospital type						
Teaching hospital (ref)	—	—	—	—	—	
General hospital	1.04	0.92-1.17	0.6	0.97	0.84-1.12	0.7
Ethnicity						
White (ref)	—	—	—	—	—	—
Nonwhite	0.84	0.70-1.02	0.074	0.89	0.72-1.11	0.3
Index of multiple deprivation						
1—most deprived (ref)	—	—	—	—	—	
2	1.02	0.85-1.22	0.8	1.17	0.94-1.44	0.2
3	0.88	0.74-1.05	0.2	0.94	0.77-1.16	0.6
4	0.94	0.79-1.11	0.4	0.99	0.82-1.21	>0.9
5—least deprived	0.87	0.73-1.03	0.10	0.94	0.77-1.15	0.6

CI, confidence interval; HR, hazard ratio.

a Fisher's exact test; Wilcoxon rank sum test; Pearson's Chi-squared test.

## Discussion

This study, including 4619 patients, identified a median TTC of 38 days overall (IQR 30-49 days), with 61% of patients receiving adjuvant chemotherapy within ≤6 weeks of surgery. Prolonged TTC ≤6 weeks was associated with improved 5-year overall survival for patients receiving IDS; however, TTC ≤6 weeks was not associated with improved survival for patients treated with primary surgery.

Similar findings have been observed in studies in the USA, where 67% of patients were treated within ≤6 weeks of debulking surgery.^27^ The median TTC of 38 days in England was greater than that of many other countries, including China (15 days, IQR 4-62 days),^28^ Germany^19^^,^^29^ and Sweden (30 days, IQR 28-37 days),^30^ and was comparable with France (43 days, IQR 36-56 days)^12^ and Italy (45 days, IQR 38-58 days).^31^ The median TTC of 38 days is potentially of concern, as a majority of patients exceeded the 4-week threshold recommended by ESMO guidelines.^23^ Prolonged waits for adjuvant chemotherapy may be related to the documented poorer performance of the UK compared with other high income countries such as Australia, Canada and Norway, in terms of cancer survival.^32^ This analysis also found a greater proportion of patients taking >6 weeks to initiate adjuvant SACT than reported by other authors for the English ovarian cancer population.^33^

TTC >6 weeks from interval surgery was associated with poorer survival. The HR of 1.18 (95% CI 1.06-1.33) indicates a small (but significant) association of TTC >6 weeks from interval surgery to chemotherapy; however, this was not found in the primary setting. Similar findings have been reported from analysis of the United States-based Surveillance, Epidemiology and End Results (SEER) database, reporting an HR of 1.11 (95% CI 1.00-1.20) for TTC >6 weeks for elderly patients with advanced-stage ovarian cancer; however, the effect size was borderline for statistical significance. In early-stage epithelial ovarian cancer, a >42-day TTC period has been associated with 4% greater risk of 5-year mortality.^27^

In the primary setting, TTC >6 weeks was not associated with poorer 5-year overall survival. This finding concords with other work reporting no association between TTC and overall survival in ovarian cancer.^12^^,^^34^ This may reflect a smaller impact of chemotherapy weeks on survival in this cohort, given that patients undergoing primary surgery are generally fitter with less extensive disease, and experience better survival outcomes than interval surgery patients. This was shown in our study, with median survival 8.2 months greater in the PDS cohort, likely due to more patients with high grade disease being treated with (neo)adjuvant chemotherapy and interval surgery.^35^

Other studies observing association of prolonged TTC with poorer survival have reported HR values of 1.53 (95% CI 1.01-2.32) for TTC >14 days versus <14 days^36^ and HR 1.09 (95% CI 1.01-1.18, *P* = 0.04)^29^ when analysing TTC as a continuous variable (per day of delay) have been reported. Our findings are concordant with Timmermans et al.,^9^ however these authors reported a larger effect size (HR 1.43, 95% CI 1.09-1.88) for patients with TTC >37 days, significant in both primary and interval surgery settings. Conflicting evidence has also been presented with a meta-analysis of randomised controlled trials and observational data^37^ concluding TTC was not positively associated with survival outcomes in ovarian cancer patients, instead suggesting that undergoing optimal debulking surgery was a more significant prognostic factor relative to survival. This is supported by Timmermans et al., who reported an association of TTC period with survival only in patients with no residual disease following surgery.^9^ Definitions of delayed TTC also differ, with >40 days used as the greatest threshold value, similar to the median TTC observed in our analysis.

Our study supports other research demonstrating associations of TTC >6 weeks with poorer overall survival in specific settings; however, this was only observed in patients receiving interval surgery in our analysis. We have demonstrated that these findings are relevant and have external validity, studying a large national cohort representative of the English ovarian cancer population over a 5-year period.

Geographical disparities in rates of surgical resection identified in the 2016-2018 ovarian cancer audit.^38^ In our analysis, median TTC in the North West was 8 days greater than in London, and treatment in London-based centres was positively associated with improved survival in the IDS cohort. This may be due to the fact that there are more specialist centres for the treatment of ovarian cancer in London, and may be indicative of geographical differences in quality of care. Greater socioeconomic deprivation was associated with poorer survival in the primary surgery cohort, suggesting this factor is associated with survival outcomes in this group, corroborating numerous studies showing association between socioeconomic deprivation and survival outcomes in the UK.^39^, ^40^, ^41^ Despite this, our analysis did not show that patients from deprived backgrounds were more likely to wait longer for adjuvant treatment. Reassuringly, differences in TTC between ethnicity groups were not observed.

The observational nature of this study and use of population-level data imposed several limitations to our analysis. Residual disease status was unavailable in the data available. The significance of residual disease has been discussed by other authors examining the impact of TTC >6 weeks, who have highlighted the impact of residual disease as a significant prognostic factor.^42^ Data on the extent of surgery (e.g. the need for multivisceral resection) and post-operative complications were not available. PS, based on commonly used ECOG score, was highlighted as an important prognostic factor in the planning of this study. PS was available for 77% of patients. To account for this, we presented complete-case analyses for patients with recorded PS, finding similar results to analysis of the whole study cohort, where PS was not included as an explanatory covariate. This method, producing similar results, suggests the analytical approach was robust in the presence of missing PS. We acknowledge that a significant proportion of patients had a missing PS, and that there may be the potential for subjectivity with this measure; PS derived from the ECOG score, however, is a commonly used measure of patient fitness in oncology, using specifically defined classifications. We therefore considered this a valid metric by which to conduct the complete-case analysis, comparing these findings with the primary analysis (PS), with results showing similar effects of TTC both in the presence and absence of PS included in the regression model.

We also recognise that bevacizumab maintenance therapy following first-line chemotherapy is an important factor impacting survival, incorporating this into survival analyses as a baseline covariate. This study did not investigate underlying reasons for delays in adjuvant chemotherapy, which can be attributed to various tumour- and patient-specific factors such as advanced age, comorbidities, and frailty, which are associated with delays in adjuvant treatment. Tumour and operative factors are also associated with delays to adjuvant chemotherapy,^43^ including extended hospital stays, residual disease, post-operative complications, bowel resection or need for re-laparotomy.^44^ Consequently, a definitive causal relationship between delay and poorer outcomes cannot be established due the observational nature of this study.

Despite the limitations of observational research, this is the first study to investigate TTC in the English ovarian cancer population, providing a benchmark for future research. It contributes to the growing body of evidence across various cancer types emphasising the importance of minimising treatment delays to enhance patient outcomes and ensure an optimal patient experience.

## Conclusions

This study demonstrated median TTC for patients with ovarian cancer in England was 38 days, identifying regional disparities in the number of patients treated within ≤6 weeks of surgery, with London significantly outperforming other regions. Reassuringly, this analysis did not identify significant inequalities relative to patient’s ethnicity or socioeconomic status. TTC ≤6 weeks was associated with improved survival for patients treated with interval surgery, but this was not the case for patients receiving primary surgery.

## Supplementary data

Supplementary Table 3

Supplementary checklist
