# Supplementary material for: Time to adjuvant chemotherapy and overall survival in advanced-stage ovarian cancer patients in England: a population-based retrospective cohort study
Source: ESMO Real World Data Digit Oncol. 2025 Apr 28;8:100143. doi: 10.1016/j.esmorw.2025.100143 (PMC12836497; doi:10.1016/j.esmorw.2025.100143)

Supplementary Figure 1: Boxplot comparison of time to adjuvant chemotherapy in days between NHS commissioning regions of England.

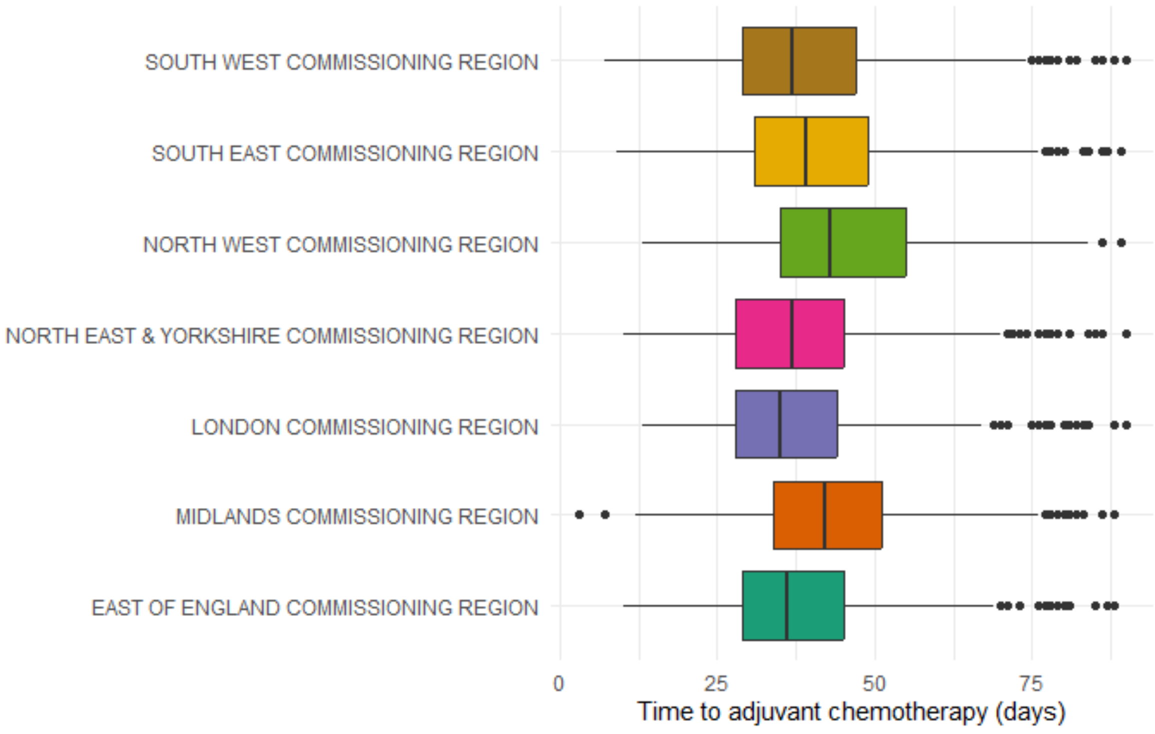

Supplement: Supplementary Figure 1 [file mmc1.pdf]
