# Supplementary material for: Time to adjuvant chemotherapy and overall survival in advanced-stage ovarian cancer patients in England: a population-based retrospective cohort study
Source: ESMO Real World Data Digit Oncol. 2025 Apr 28;8:100143. doi: 10.1016/j.esmorw.2025.100143 (PMC12836497; doi:10.1016/j.esmorw.2025.100143)

Supplementary Figure 2: Boxplot comparison of time to adjuvant chemotherapy in days between age categories (<60, 60-70, <70 years of age).

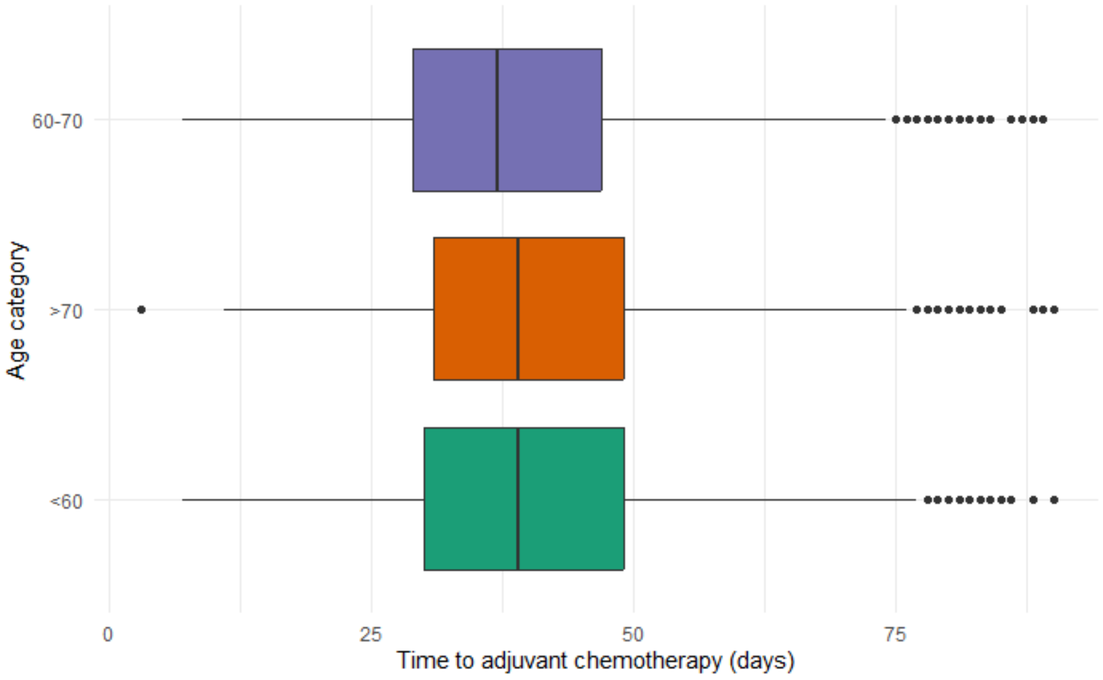

Supplement: Supplementary Figure 2 [file mmc2.pdf]
