# Supplementary material for: Time to adjuvant chemotherapy and overall survival in advanced-stage ovarian cancer patients in England: a population-based retrospective cohort study
Source: ESMO Real World Data Digit Oncol. 2025 Apr 28;8:100143. doi: 10.1016/j.esmorw.2025.100143 (PMC12836497; doi:10.1016/j.esmorw.2025.100143)

Supplementary Figure 4: Boxplot comparison of time to adjuvant chemotherapy in days by surgical modality (PDS: primary debulking surgery, IDS: interval debulking surgery).

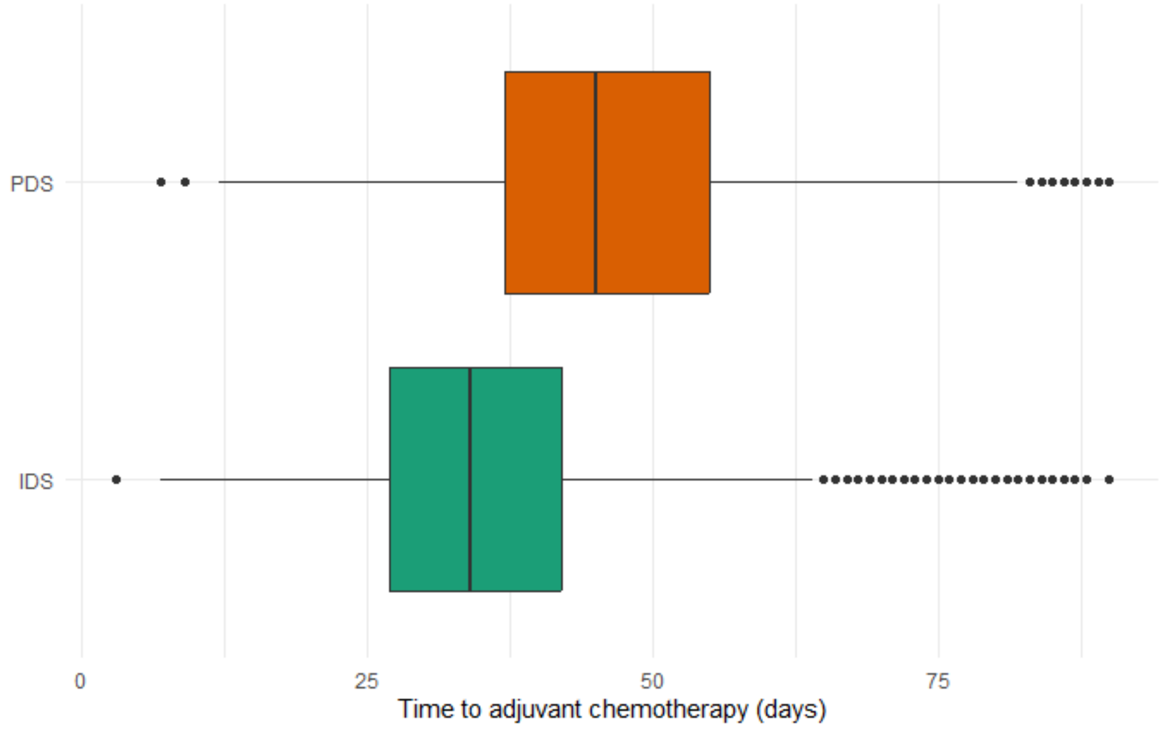

Supplement: Supplementary Figure 4 [file mmc4.pdf]
