# Supplementary material for: Time to adjuvant chemotherapy and overall survival in advanced-stage ovarian cancer patients in England: a population-based retrospective cohort study
Source: ESMO Real World Data Digit Oncol. 2025 Apr 28;8:100143. doi: 10.1016/j.esmorw.2025.100143 (PMC12836497; doi:10.1016/j.esmorw.2025.100143)
Supplement: Supplementary Material [file mmc5.docx]

# SUPPLEMENTAL MATERIAL

# Supplemental methods

# Predictors - Blood test values

Blood test values included in the extended model: (i.e. hemoglobin, leucocytes, thrombocytes, mean corpuscular volume (MCV), glomerular filtration rate (GFR), urea, calcium, sodium, potassium, glucose, C reactive protein (CRP), alanine aminotransferase (ALAT), alkaline phosphatase (AF), aspartate aminotransferase (ASAT) and gamma−glutamyl transferase (gamma-GT))

# Sample size calculation

Developing a reliable prediction model requires a development dataset with a large enough sample size. The effective sample size for binary outcomes is defined as the minimum number of events and non-events. To increase the potential for developing a robust prediction model, the sample size of the development set should be large enough to minimize overfitting and to calculate precise predictions. According to the criteria proposed by Riley et al, the sample size for binary outcomes should be large enough to [1] target small overfitting defined by an expected shrinkage of predictor effects by 10% or less; [2] target a small absolute difference of 0.05 in the model's apparent and adjusted Nagelkerke's R-squared value and [3] precisely estimate the (within +/- 0.05) average outcome risk in the population for a key timepoint of interest for prediction. In our study we had a total of 103 candidate predictor parameters, including all levels of categorical variables and potential non-linear transformations for continuous variables. According to literature the probability of hospitalization for cancer patients presenting at the ED is estimated to be 50%. Additionally, due to the absence of existing prediction models, the expected value of Nagelkerke's R-squared was set to 0.15. Minimum sample size calculations were performed using R-package pmsampsize.

# Supplemental results

**Baseline model**

Linear predictor (lp) *= 5.1335 + 0.0112 * Age − 0.3015* Sex_Female + 1.1816 ∗ Mode of transportation_Ambulance + 0.3886 ∗ Primary malignancy + 0.2449 ∗ Metastasis registered + 0.8179 ∗ Symptoms + 0.0171*HR − 0.0236 * (HR` −120) − 0.0228 * DBP + 0.0200 * (DBP −80) − 0.1717 ∗ Temperature + 0.8981 ∗ (Temperature’−37) + 0.0390 * VAS + 0.0555 ∗ (VAS’ −2)*

Probability of hospitalization *=1/(1+exp(-lp))*

**Extended model**

Linear predictor (lp) = *27.8857 + 1.1589 ∗ Mode of transportation_Ambulance + 0.3110 ∗ Primary malignancy+ 0.3343 ∗ Metastasis registered + 0.8415 ∗ Symptoms at presentation − 0.1388 ∗ Temperature+ 0.8397 ∗ (Temperature’−37) + 0.0148 * VAS + 0.0613 ∗ (VAS’ – 2) + 0.0408 ∗ Leukocytes − 0.0373 ∗ (Leucocytes′ – 18) + 0.0123 ∗ CRP − 0.0087 ∗ (CRP′ – 46) − 0.1782 ∗ Sodium + 0.1421 ∗ (Sodium′ – 130) − 0.5122 ∗ Potassium + 0.5012 ∗ (Potassium′ – 4.2) + 0.5253 ∗ log(Urea)*

Probability of hospitalization =1/(1+exp(-lp))

**Supplemental Table 1.** Minimum sample size calculations for the development of a prediction model, according to the criteria proposed by Riley et al. Calculations include an outcome probability of 50%, a candidate predictor parameter number of 103, a targeted shrinkage of 0.9 and targeted Nagelkerke's R-squared of 0.15.

|  | Calculated required sample size | Targeted shrinkage | Number of candidate parameters | Cox-Snell R-squared | Maximum  R-squared | Targeted Nagelkerke's R-squared | Calculated event per candidate predictor parameter (EPP) |
| --- | --- | --- | --- | --- | --- | --- | --- |
| **Criteria I** | **7751** | 0.9 | 103 | 0.112 | 0.75 | 0.15 | 37.6 |
| **Criteria II** | **2536** | 0.9 | 103 | 0.112 | 0.75 | 0.15 | 12.3 |
| **Criteria III** | **385** | 0.9 | 103 | 0.112 | 0.75 | 0.15 | 1.9 |

**Supplemental table.2** Symptoms at ED presentation according to primary cancer type

| **Symptoms at ED arrival** | **Overall**, N = 7,284^1^ | **Head and neck cancer**, N = 580^1^ | **Lung cancer**, N = 856^1^ | **Breast cancer**,  N = 410^1^ | **Gallbladder, liver and pancreatic cancer**, N = 577^1^ | **Colorectal cancer**, N = 467^1^ | **Other Gastrointestinal tumors**, N = 189^1^ | **Melanoma**, N = 593^1^ | **Cancer of bone and connective tissue**, N = 182^1^ | **Female reproductive**, N = 498^1^ | **Stomach and esophagus cancer**, N = 340^1^ | **Central nerve system tumor**, N = 423^1^ | **Hematological cancer**, N = 1,246^1^ | **Urinary tract cancer**, N = 632^1^ | **Male reproductive cancer**, N = 85^1^ | **Prostate cancer**, N = 100^1^ | **Unspecified malignancy**, N = 106^1^ |  |
| --- | --- | --- | --- | --- | --- | --- | --- | --- | --- | --- | --- | --- | --- | --- | --- | --- | --- | --- |
| Abdominal pain | 783 (11%) | 27 (4.7%) | 37 (4.3%) | 23 (5.6%) | 136 (24%) | 110 (24%) | 45 (24%) | 32 (5.4%) | 27 (15%) | 90 (18%) | 49 (14%) | 15 (3.5%) | 79 (6.3%) | 82 (13%) | 1 (1.2%) | 9 (9.0%) | 21 (20%) |  |
| Abnormalities of breathing | 876 (12%) | 138 (24%) | 233 (27%) | 41 (10%) | 28 (4.9%) | 21 (4.5%) | 15 (7.9%) | 53 (8.9%) | 24 (13%) | 33 (6.6%) | 56 (16%) | 25 (5.9%) | 139 (11%) | 51 (8.1%) | 7 (8.2%) | 7 (7.0%) | 5 (4.7%) |  |
| Abscess or other local infection | 229 (3.1%) | 32 (5.5%) | 11 (1.3%) | 17 (4.1%) | 10 (1.7%) | 21 (4.5%) | 4 (2.1%) | 30 (5.1%) | 19 (10%) | 27 (5.4%) | 7 (2.1%) | 10 (2.4%) | 21 (1.7%) | 8 (1.3%) | 3 (3.5%) | 3 (3.0%) | 6 (5.7%) |  |
| Cardiac dysrhythmia | 227 (3.1%) | 15 (2.6%) | 31 (3.6%) | 26 (6.3%) | 18 (3.1%) | 11 (2.4%) | 1 (0.5%) | 20 (3.4%) | 6 (3.3%) | 13 (2.6%) | 11 (3.2%) | 4 (0.9%) | 44 (3.5%) | 15 (2.4%) | 2 (2.4%) | 7 (7.0%) | 3 (2.8%) |  |
| Collapse | 166 (2.3%) | 15 (2.6%) | 16 (1.9%) | 8 (2.0%) | 14 (2.4%) | 6 (1.3%) | 3 (1.6%) | 21 (3.5%) | 2 (1.1%) | 4 (0.8%) | 11 (3.2%) | 18 (4.3%) | 30 (2.4%) | 13 (2.1%) | 1 (1.2%) | 1 (1.0%) | 3 (2.8%) |  |
| Cognitive and psychiatric complaints | 132 (1.8%) | 7 (1.2%) | 25 (2.9%) | 6 (1.5%) | 13 (2.3%) | 6 (1.3%) | 5 (2.6%) | 14 (2.4%) | 1 (0.5%) | 4 (0.8%) | 4 (1.2%) | 19 (4.5%) | 17 (1.4%) | 6 (0.9%) | 2 (2.4%) | 3 (3.0%) | 0 (0%) |  |
| Dermatological and allergic complaints | 96 (1.3%) | 4 (0.7%) | 7 (0.8%) | 7 (1.7%) | 3 (0.5%) | 4 (0.9%) | 2 (1.1%) | 12 (2.0%) | 2 (1.1%) | 8 (1.6%) | 1 (0.3%) | 7 (1.7%) | 33 (2.6%) | 6 (0.9%) | 0 (0%) | 0 (0%) | 0 (0%) |  |
| Disorders of urinary system | 207 (2.8%) | 7 (1.2%) | 3 (0.4%) | 5 (1.2%) | 0 (0%) | 22 (4.7%) | 3 (1.6%) | 10 (1.7%) | 4 (2.2%) | 36 (7.2%) | 0 (0%) | 0 (0%) | 13 (1.0%) | 88 (14%) | 2 (2.4%) | 12 (12%) | 2 (1.9%) |  |
| Extremity complaints | 345 (4.7%) | 19 (3.3%) | 40 (4.7%) | 34 (8.3%) | 11 (1.9%) | 15 (3.2%) | 5 (2.6%) | 25 (4.2%) | 17 (9.3%) | 19 (3.8%) | 5 (1.5%) | 18 (4.3%) | 87 (7.0%) | 32 (5.1%) | 6 (7.1%) | 10 (10%) | 2 (1.9%) |  |
| Fever | 665 (9.1%) | 28 (4.8%) | 74 (8.6%) | 31 (7.6%) | 82 (14%) | 38 (8.1%) | 18 (9.5%) | 54 (9.1%) | 20 (11%) | 45 (9.0%) | 20 (5.9%) | 23 (5.4%) | 137 (11%) | 65 (10%) | 15 (18%) | 9 (9.0%) | 6 (5.7%) |  |
| Gastro-intestinal bleeding | 128 (1.8%) | 8 (1.4%) | 10 (1.2%) | 2 (0.5%) | 22 (3.8%) | 22 (4.7%) | 5 (2.6%) | 11 (1.9%) | 1 (0.5%) | 13 (2.6%) | 9 (2.6%) | 3 (0.7%) | 13 (1.0%) | 7 (1.1%) | 0 (0%) | 0 (0%) | 2 (1.9%) |  |
| Headache | 250 (3.4%) | 16 (2.8%) | 38 (4.4%) | 14 (3.4%) | 0 (0%) | 8 (1.7%) | 2 (1.1%) | 31 (5.2%) | 2 (1.1%) | 6 (1.2%) | 3 (0.9%) | 88 (21%) | 20 (1.6%) | 13 (2.1%) | 3 (3.5%) | 4 (4.0%) | 2 (1.9%) |  |
| Insult | 85 (1.2%) | 5 (0.9%) | 14 (1.6%) | 2 (0.5%) | 0 (0%) | 1 (0.2%) | 1 (0.5%) | 8 (1.3%) | 0 (0%) | 1 (0.2%) | 2 (0.6%) | 41 (9.7%) | 4 (0.3%) | 5 (0.8%) | 1 (1.2%) | 0 (0%) | 0 (0%) |  |
| Malaise | 1,323 (18%) | 107 (18%) | 131 (15%) | 74 (18%) | 100 (17%) | 62 (13%) | 34 (18%) | 97 (16%) | 27 (15%) | 72 (14%) | 80 (24%) | 53 (13%) | 353 (28%) | 83 (13%) | 17 (20%) | 16 (16%) | 17 (16%) |  |
| Neurologic deficit | 160 (2.2%) | 8 (1.4%) | 29 (3.4%) | 9 (2.2%) | 2 (0.3%) | 5 (1.1%) | 3 (1.6%) | 18 (3.0%) | 1 (0.5%) | 4 (0.8%) | 1 (0.3%) | 35 (8.3%) | 26 (2.1%) | 17 (2.7%) | 0 (0%) | 1 (1.0%) | 1 (0.9%) |  |
| Nonspecific chest pain | 237 (3.3%) | 7 (1.2%) | 36 (4.2%) | 23 (5.6%) | 20 (3.5%) | 15 (3.2%) | 2 (1.1%) | 28 (4.7%) | 1 (0.5%) | 11 (2.2%) | 7 (2.1%) | 5 (1.2%) | 42 (3.4%) | 26 (4.1%) | 5 (5.9%) | 4 (4.0%) | 5 (4.7%) |  |
| Pain | 395 (5.4%) | 23 (4.0%) | 53 (6.2%) | 37 (9.0%) | 39 (6.8%) | 22 (4.7%) | 16 (8.5%) | 18 (3.0%) | 10 (5.5%) | 30 (6.0%) | 15 (4.4%) | 17 (4.0%) | 42 (3.4%) | 53 (8.4%) | 7 (8.2%) | 3 (3.0%) | 10 (9.4%) |  |
| Septicemia | 102 (1.4%) | 5 (0.9%) | 7 (0.8%) | 5 (1.2%) | 8 (1.4%) | 5 (1.1%) | 8 (4.2%) | 8 (1.3%) | 2 (1.1%) | 9 (1.8%) | 6 (1.8%) | 1 (0.2%) | 22 (1.8%) | 9 (1.4%) | 2 (2.4%) | 4 (4.0%) | 1 (0.9%) |  |
| Trauma | 105 (1.4%) | 7 (1.2%) | 5 (0.6%) | 9 (2.2%) | 2 (0.3%) | 4 (0.9%) | 1 (0.5%) | 16 (2.7%) | 3 (1.6%) | 4 (0.8%) | 3 (0.9%) | 15 (3.5%) | 22 (1.8%) | 10 (1.6%) | 2 (2.4%) | 1 (1.0%) | 1 (0.9%) |  |
| Vomiting and/or diarrhea | 315 (4.3%) | 18 (3.1%) | 29 (3.4%) | 14 (3.4%) | 46 (8.0%) | 48 (10%) | 12 (6.3%) | 21 (3.5%) | 1 (0.5%) | 18 (3.6%) | 23 (6.8%) | 9 (2.1%) | 41 (3.3%) | 18 (2.8%) | 2 (2.4%) | 3 (3.0%) | 12 (11%) |  |
| Wound problems | 174 (2.4%) | 14 (2.4%) | 8 (0.9%) | 11 (2.7%) | 9 (1.6%) | 13 (2.8%) | 0 (0%) | 44 (7.4%) | 10 (5.5%) | 13 (2.6%) | 6 (1.8%) | 3 (0.7%) | 22 (1.8%) | 12 (1.9%) | 4 (4.7%) | 1 (1.0%) | 4 (3.8%) |  |
| Other | 218 (3.0%) | 60 (10%) | 15 (1.8%) | 7 (1.7%) | 9 (1.6%) | 6 (1.3%) | 0 (0%) | 18 (3.0%) | 0 (0%) | 34 (6.8%) | 15 (4.4%) | 11 (2.6%) | 30 (2.4%) | 7 (1.1%) | 3 (3.5%) | 1 (1.0%) | 2 (1.9%) |  |
| Unknown | 66 (0.9%) | 10 (1.7%) | 4 (0.5%) | 5 (1.2%) | 5 (0.9%) | 2 (0.4%) | 4 (2.1%) | 4 (0.7%) | 2 (1.1%) | 4 (0.8%) | 6 (1.8%) | 3 (0.7%) | 9 (0.7%) | 6 (0.9%) | 0 (0%) | 1 (1.0%) | 1 (0.9%) |  |
| ^1^n (%) | | | | | | | | | | | | | | | | | | |

**Supplemental table 3.** Treatment, metastasis and presence of multiple tumors according to primary tumor type

|  | | | **Multiple tumours** | | | | **Metastasis registered** | | | | **Systemic treatment** | | |
| --- | --- | --- | --- | --- | --- | --- | --- | --- | --- | --- | --- | --- | --- |
| **Primary cancer type** | **Overall**, N = 7,284 | **One malignancy diagnosed**, N = 6,279 | | **>1 malignancy diagnosed**, N = 1,005 | **No metastasis registered**, N = 6,286 | **Metastasis registered**, N = 998 | | **No systemic treatment**, N = 4,157 | **Chemoimmunotherapy**, N = 320 | **Chemotherapy**, N = 1,505 | | **Immunotherapy**, N = 1,224 | **Hormonal Therapy**, N = 78 |
| Head and neck cancer | 580 (8.0%) | 466 (7.4%) | | 114 (11%) | 549 (8.7%) | 31 (3.1%) | | 443 (11%) | 6 (1.9%) | 83 (5.5%) | | 48 (3.9%) | 0 (0%) |
| Lungcancer | 856 (12%) | 757 (12%) | | 99 (9.9%) | 661 (11%) | 195 (20%) | | 340 (8.2%) | 102 (32%) | 229 (15%) | | 184 (15%) | 1 (1.3%) |
| Breastcancer | 410 (5.6%) | 356 (5.7%) | | 54 (5.4%) | 317 (5.0%) | 93 (9.3%) | | 164 (3.9%) | 20 (6.3%) | 145 (9.6%) | | 24 (2.0%) | 57 (73%) |
| Gallbladder, liver and pancreatic cancer | 577 (7.9%) | 537 (8.6%) | | 40 (4.0%) | 565 (9.0%) | 12 (1.2%) | | 428 (10%) | 3 (0.9%) | 117 (7.8%) | | 29 (2.4%) | 0 (0%) |
| Colorectal cancer | 467 (6.4%) | 426 (6.8%) | | 41 (4.1%) | 334 (5.3%) | 133 (13%) | | 299 (7.2%) | 17 (5.3%) | 128 (8.5%) | | 22 (1.8%) | 1 (1.3%) |
| Other Gastrointestinal tumors | 189 (2.6%) | 154 (2.5%) | | 35 (3.5%) | 168 (2.7%) | 21 (2.1%) | | 135 (3.2%) | 1 (0.3%) | 45 (3.0%) | | 5 (0.4%) | 3 (3.8%) |
| Melanoma | 593 (8.1%) | 468 (7.5%) | | 125 (12%) | 484 (7.7%) | 109 (11%) | | 376 (9.0%) | 21 (6.6%) | 34 (2.3%) | | 158 (13%) | 4 (5.1%) |
| Cancer of bone and connective tissue | 182 (2.5%) | 131 (2.1%) | | 51 (5.1%) | 166 (2.6%) | 16 (1.6%) | | 106 (2.5%) | 7 (2.2%) | 33 (2.2%) | | 36 (2.9%) | 0 (0%) |
| Female reproductive | 498 (6.8%) | 407 (6.5%) | | 91 (9.1%) | 471 (7.5%) | 27 (2.7%) | | 374 (9.0%) | 6 (1.9%) | 92 (6.1%) | | 23 (1.9%) | 3 (3.8%) |
| Stomach and esophagus cancer | 340 (4.7%) | 310 (4.9%) | | 30 (3.0%) | 307 (4.9%) | 33 (3.3%) | | 186 (4.5%) | 1 (0.3%) | 151 (10%) | | 1 (<0.1%) | 1 (1.3%) |
| Central nerve system tumor | 423 (5.8%) | 359 (5.7%) | | 64 (6.4%) | 396 (6.3%) | 27 (2.7%) | | 248 (6.0%) | 3 (0.9%) | 141 (9.4%) | | 27 (2.2%) | 4 (5.1%) |
| Hematological cancer | 1,246 (17%) | 1,155 (18%) | | 91 (9.1%) | 1,033 (16%) | 213 (21%) | | 409 (9.8%) | 117 (37%) | 171 (11%) | | 549 (45%) | 0 (0%) |
| Urinary tract cancer | 632 (8.7%) | 502 (8.0%) | | 130 (13%) | 567 (9.0%) | 65 (6.5%) | | 442 (11%) | 15 (4.7%) | 67 (4.5%) | | 107 (8.7%) | 1 (1.3%) |
| Male reproductive cancer | 85 (1.2%) | 74 (1.2%) | | 11 (1.1%) | 77 (1.2%) | 8 (0.8%) | | 45 (1.1%) | 0 (0%) | 37 (2.5%) | | 3 (0.2%) | 0 (0%) |
| Prostate cancer | 100 (1.4%) | 71 (1.1%) | | 29 (2.9%) | 92 (1.5%) | 8 (0.8%) | | 67 (1.6%) | 1 (0.3%) | 26 (1.7%) | | 3 (0.2%) | 3 (3.8%) |
| Unspecified malignancy | 106 (1.5%) | 106 (1.7%) | | 0 (0%) | 99 (1.6%) | 7 (0.7%) | | 95 (2.3%) | 0 (0%) | 6 (0.4%) | | 5 (0.4%) | 0 (0%) |

**Supplemental table 4.** Baseline model: Univariable and multivariable associations between predictors and hospitalization OR with 95% CIs for separate variables (columns “Univariable model”) and for a model with all available predictors (columns “Multivariable model)

| **Predictor** | **Univariable analysis** | | | | | **Multivariable analysis** | | | | |
| --- | --- | --- | --- | --- | --- | --- | --- | --- | --- | --- |
|  | **OR** | **95% CI** | | | **Wald** | **OR** | **95% CI** | | | **Wald** |
| **Age, years (54 vs 71)** | 1.25 | 1.17 | - | 1.33 | 47 | 1.26 | 1.14 | - | 1.39 | 24 |
| **Sex (ref. male)** | 0.81 | 0.74 | - | 0.90 | 15 | 0.78 | 0.68 | - | 0.89 | 13 |
| **Ambulance transportation (ref. own transportation)** | 3.64 | 3.21 | - | 4.13 | 401 | 3.40 | 2.93 | - | 3.94 | 261 |
| **Primary malignancy (ref. hematological cancer)** |  |  |  |  | 105 |  |  |  |  | 44 |
| Head and neck cancer | 1.11 | 0.89 | - | 1.39 |  | 1.11 | 0.85 | - | 1.44 |  |
| Lung cancer | 1.45 | 1.19 | - | 1.76 |  | 1.33 | 1.05 | - | 1.67 |  |
| Breast cancer | 0.60 | 0.47 | - | 0.77 |  | 0.75 | 0.55 | - | 1.02 |  |
| Hepatopancreatobiliary cancer | 1.77 | 1.42 | - | 2.22 |  | 1.57 | 1.20 | - | 2.06 |  |
| Colorectal cancer | 1.52 | 1.20 | - | 1.93 |  | 1.49 | 1.12 | - | 1.98 |  |
| Non-specified gastrointestinal tumors | 2.03 | 1.42 | - | 2.90 |  | 1.61 | 1.07 | - | 2.45 |  |
| Melanoma | 0.90 | 0.73 | - | 1.12 |  | 0.96 | 0.74 | - | 1.24 |  |
| Cancer of bone and connective tissue | 1.19 | 0.84 | - | 1.67 |  | 1.29 | 0.86 | - | 1.93 |  |
| Female reproductive | 0.99 | 0.78 | - | 1.24 |  | 1.43 | 1.07 | - | 1.92 |  |
| Stomach and esophagus cancer | 0.96 | 0.74 | - | 1.25 |  | 0.94 | 0.69 | - | 1.28 |  |
| Central nerve system tumor | 0.98 | 0.77 | - | 1.25 |  | 1.10 | 0.81 | - | 1.50 |  |
| Urinary tract cancer | 1.22 | 0.98 | - | 1.50 |  | 1.26 | 0.98 | - | 1.63 |  |
| Male reproductive cancer | 0.77 | 0.48 | - | 1.25 |  | 1.00 | 0.56 | - | 1.78 |  |
| Prostate cancer | 0.96 | 0.61 | - | 1.50 |  | 0.74 | 0.43 | - | 1.27 |  |
| Unspecified malignancy | 1.10 | 0.71 | - | 1.71 |  | 1.38 | 0.82 | - | 2.31 |  |
| **Multiple tumors (One vs. >1 malignancy)** | 1.19 | 1.02 | - | 1.37 | 5 | 1.03 | 0.86 | - | 1.22 | 0 |
| **Metastasis registered (ref. no metastasis)** | 1.28 | 1.10 | - | 1.49 | 11 | 1.35 | 1.13 | - | 1.61 | 11 |
| **Systemic treatment (ref. no treatment)** |  |  |  |  | 10 |  |  |  |  | 7 |
| Chemoimmunotherapy | 0.81 | 0.62 | - | 1.06 |  | 0.71 | 0.52 | - | 0.97 |  |
| Chemotherapy | 0.92 | 0.81 | - | 1.05 |  | 0.88 | 0.75 | - | 1.03 |  |
| Immunotherapy | 0.89 | 0.77 | - | 1.02 |  | 0.86 | 0.72 | - | 1.02 |  |
| Hormonal Therapy | 0.54 | 0.33 | - | 0.88 |  | 0.87 | 0.48 | - | 1.58 |  |
| **Symptoms at presentation (ref. malaise)** |  |  |  |  | 436 |  |  |  |  | 272 |
| Abdominal pain | 0.87 | 0.72 | - | 1.06 |  | 0.69 | 0.55 | - | 0.87 |  |
| Abnormalities of breathing | 1.18 | 0.97 | - | 1.43 |  | 0.98 | 0.78 | - | 1.22 |  |
| Abscess or other local infection | 0.26 | 0.18 | - | 0.37 |  | 0.30 | 0.21 | - | 0.44 |  |
| Cardiac dysrhythmia | 0.62 | 0.46 | - | 0.86 |  | 0.35 | 0.24 | - | 0.52 |  |
| Collapse | 1.25 | 0.87 | - | 1.79 |  | 0.85 | 0.56 | - | 1.29 |  |
| Cognitive and psychiatric complaints | 2.22 | 1.41 | - | 3.49 |  | 2.01 | 1.22 | - | 3.29 |  |
| Dermatological and allergic complaints | 0.47 | 0.30 | - | 0.74 |  | 0.55 | 0.33 | - | 0.90 |  |
| Disorders of urinary system | 0.39 | 0.28 | - | 0.55 |  | 0.39 | 0.26 | - | 0.56 |  |
| Extremity complaints | 0.32 | 0.24 | - | 0.42 |  | 0.26 | 0.19 | - | 0.35 |  |
| Fever | 1.83 | 1.47 | - | 2.28 |  | 0.93 | 0.71 | - | 1.21 |  |
| Gastro-intestinal bleeding | 2.11 | 1.35 | - | 3.31 |  | 1.68 | 1.03 | - | 2.74 |  |
| Headache | 1.21 | 0.89 | - | 1.64 |  | 1.11 | 0.78 | - | 1.57 |  |
| Insult | 1.13 | 0.68 | - | 1.89 |  | 0.63 | 0.35 | - | 1.12 |  |
| Neurologic deficit | 1.25 | 0.87 | - | 1.82 |  | 0.95 | 0.62 | - | 1.44 |  |
| Nonspecific chest pain | 0.45 | 0.33 | - | 0.61 |  | 0.28 | 0.19 | - | 0.40 |  |
| Pain | 1.06 | 0.83 | - | 1.36 |  | 0.84 | 0.63 | - | 1.12 |  |
| Septicemia | 8.67 | 3.74 | - | 20.08 |  | 1.97 | 0.81 | - | 4.82 |  |
| Trauma | 1.12 | 0.72 | - | 1.73 |  | 0.67 | 0.41 | - | 1.10 |  |
| Vomiting and/or diarrhea | 1.96 | 1.46 | - | 2.63 |  | 1.99 | 1.43 | - | 2.76 |  |
| Wound problems | 0.23 | 0.16 | - | 0.34 |  | 0.25 | 0.16 | - | 0.39 |  |
| Other | 0.39 | 0.28 | - | 0.55 |  | 0.44 | 0.30 | - | 0.64 |  |
| **Heart rate (bpm) (77 vs 104)** | 1.55 | 1.42 | - | 1.70 | 87 | 1.47 | 1.30 | - | 1.67 | 41 |
| **SBP (mmHg) (121 vs 152)** | 0.75 | 0.70 | - | 0.81 | 61 | 0.92 | 0.81 | - | 1.04 | 2 |
| **DBP (mmHg) (71 vs 91)** | 0.72 | 0.67 | - | 0.78 | 74 | 0.84 | 0.74 | - | 0.95 | 12 |
| **Saturation (%) (95 vs. 98)** | 0.88 | 0.83 | - | 0.94 | 16 | 0.93 | 0.85 | - | 1.03 | 4 |
| **Temperature (°C) (36 vs 38)** | 1.55 | 1.45 | - | 1.66 | 156 | 1.22 | 1.10 | - | 1.36 | 67 |
| **VAS pain score (0 vs 4)** | 1.18 | 1.08 | - | 1.30 | 13 | 1.51 | 1.28 | - | 1.79 | 54 |

**Supplemental table 5.** Extended model: Univariable and multivariable associations between predictors and hospitalization OR with 95% CIs for separate variables (columns “Univariable model”) and for a model with all available predictors (columns “Multivariable model)

| **Predictor** | **Univariable analysis** | | | | | **Multivariable analysis** | | | | |
| --- | --- | --- | --- | --- | --- | --- | --- | --- | --- | --- |
|  | OR | **95% CI** | | | **Wald** | **OR** | **95% CI** | | | **Wald** |
| **Age, years (54 vs 71)** | 1.25 | 1.17 | - | 1.33 | 47 | 0.95 | 0.81 | - | 1.12 | 7 |
| **Sex (ref. male)** | 0.81 | 0.74 | - | 0.90 | 15 | 0.71 | 0.55 | - | 0.92 | 3 |
| **Ambulance transportation (ref. own transportation)** | 3.64 | 3.21 | - | 4.13 | 401 | 3.11 | 2.65 | - | 3.65 | 193 |
| **Primary malignancy (ref. hematological cancer)** |  |  |  |  | 105 |  |  |  |  | 28 |
| Head and neck cancer | 1.11 | 0.89 | - | 1.39 |  | 1.31 | 0.96 | - | 1.80 |  |
| Lung cancer | 1.45 | 1.19 | - | 1.76 |  | 1.35 | 1.03 | - | 1.77 |  |
| Breast cancer | 0.60 | 0.47 | - | 0.77 |  | 0.89 | 0.62 | - | 1.27 |  |
| Hepatopancreatobiliary cancer | 1.77 | 1.42 | - | 2.22 |  | 1.19 | 0.86 | - | 1.64 |  |
| Colorectal cancer | 1.52 | 1.20 | - | 1.93 |  | 1.48 | 1.07 | - | 2.03 |  |
| Non-specified gastrointestinal tumors | 2.03 | 1.42 | - | 2.90 |  | 1.53 | 0.98 | - | 2.39 |  |
| Melanoma | 0.90 | 0.73 | - | 1.12 |  | 0.97 | 0.73 | - | 1.30 |  |
| Cancer of bone and connective tissue | 1.19 | 0.84 | - | 1.67 |  | 1.33 | 0.85 | - | 2.07 |  |
| Female reproductive | 0.99 | 0.78 | - | 1.24 |  | 1.53 | 1.10 | - | 2.13 |  |
| Stomach and esophagus cancer | 0.96 | 0.74 | - | 1.25 |  | 1.22 | 0.85 | - | 1.73 |  |
| Central nerve system tumor | 0.98 | 0.77 | - | 1.25 |  | 1.47 | 1.04 | - | 2.09 |  |
| Urinary tract cancer | 1.22 | 0.98 | - | 1.50 |  | 1.21 | 0.89 | - | 1.63 |  |
| Male reproductive cancer | 0.77 | 0.48 | - | 1.25 |  | 1.08 | 0.58 | - | 2.04 |  |
| Prostate cancer | 0.96 | 0.61 | - | 1.50 |  | 0.65 | 0.36 | - | 1.19 |  |
| Unspecified malignancy | 1.10 | 0.71 | - | 1.71 |  | 1.24 | 0.71 | - | 2.18 |  |
| **Multiple tumors (One vs. >1 malignancy)** | 1.19 | 1.02 | - | 1.37 | 5 | 1.01 | 0.84 | - | 1.22 | 0 |
| **Metastasis registered (ref. no metastasis)** | 1.28 | 1.10 | - | 1.49 | 11 | 1.40 | 1.16 | - | 1.69 | 12 |
| **Systemic treatment (ref. no treatment)** |  |  |  |  | 10 |  |  |  |  | 5 |
| Chemoimmunotherapy | 0.81 | 0.62 | - | 1.06 |  | 0.83 | 0.59 | - | 1.17 |  |
| Chemotherapy | 0.92 | 0.81 | - | 1.05 |  | 0.87 | 0.73 | - | 1.04 |  |
| Immunotherapy | 0.89 | 0.77 | - | 1.02 |  | 0.83 | 0.69 | - | 1.01 |  |
| Hormonal Therapy | 0.54 | 0.33 | - | 0.88 |  | 1.02 | 0.53 | - | 1.94 |  |
| **Symptoms at presentation (ref. malaise)** |  |  |  |  | 436 |  |  |  |  | 213 |
| Abdominal pain | 0.87 | 0.72 | - | 1.06 |  | 0.76 | 0.59 | - | 0.98 |  |
| Abnormalities of breathing | 1.18 | 0.97 | - | 1.43 |  | 1.09 | 0.85 | - | 1.38 |  |
| Abscess or other local infection | 0.26 | 0.18 | - | 0.37 |  | 0.34 | 0.22 | - | 0.52 |  |
| Cardiac dysrhythmia | 0.62 | 0.46 | - | 0.86 |  | 0.49 | 0.31 | - | 0.76 |  |
| Collapse | 1.25 | 0.87 | - | 1.79 |  | 1.00 | 0.64 | - | 1.57 |  |
| Cognitive and psychiatric complaints | 2.22 | 1.41 | - | 3.49 |  | 2.06 | 1.21 | - | 3.51 |  |
| Dermatological and allergic complaints | 0.47 | 0.30 | - | 0.74 |  | 0.60 | 0.34 | - | 1.04 |  |
| Disorders of urinary system | 0.39 | 0.28 | - | 0.55 |  | 0.38 | 0.24 | - | 0.59 |  |
| Extremity complaints | 0.32 | 0.24 | - | 0.42 |  | 0.28 | 0.20 | - | 0.40 |  |
| Fever | 1.83 | 1.47 | - | 2.28 |  | 0.95 | 0.72 | - | 1.26 |  |
| Gastro-intestinal bleeding | 2.11 | 1.35 | - | 3.31 |  | 1.93 | 1.14 | - | 3.26 |  |
| Headache | 1.21 | 0.89 | - | 1.64 |  | 1.45 | 1.01 | - | 2.09 |  |
| Insult | 1.13 | 0.68 | - | 1.89 |  | 0.78 | 0.42 | - | 1.45 |  |
| Neurologic deficit | 1.25 | 0.87 | - | 1.82 |  | 1.28 | 0.83 | - | 1.98 |  |
| Nonspecific chest pain | 0.45 | 0.33 | - | 0.61 |  | 0.39 | 0.27 | - | 0.58 |  |
| Pain | 1.06 | 0.83 | - | 1.36 |  | 0.91 | 0.67 | - | 1.24 |  |
| Septicemia | 8.67 | 3.74 | - | 20.08 |  | 1.81 | 0.72 | - | 4.53 |  |
| Trauma | 1.12 | 0.72 | - | 1.73 |  | 0.86 | 0.50 | - | 1.49 |  |
| Vomiting and/or diarrhea | 1.96 | 1.46 | - | 2.63 |  | 2.17 | 1.55 | - | 3.04 |  |
| Wound problems | 0.23 | 0.16 | - | 0.34 |  | 0.27 | 0.17 | - | 0.45 |  |
| Other | 0.39 | 0.28 | - | 0.55 |  | 0.42 | 0.27 | - | 0.64 | 9 |
| **Heart rate (bpm) (77 vs 104)** | 1.55 | 1.42 | - | 1.70 | 87 | 1.22 | 1.07 | - | 1.39 | 1 |
| **SBP (mmHg) (121 vs 152)** | 0.75 | 0.70 | - | 0.81 | 61 | 1.00 | 0.88 | - | 1.14 | 3 |
| **DBP (mmHg) (71 vs 91)** | 0.72 | 0.67 | - | 0.78 | 74 | 0.99 | 0.87 | - | 1.13 | 2 |
| **Saturation (%) (95 vs. 98)** | 0.88 | 0.83 | - | 0.94 | 16 | 0.94 | 0.85 | - | 1.04 | 51 |
| **Temperature (°C) (36 vs 38)** | 1.55 | 1.45 | - | 1.66 | 156 | 1.23 | 1.10 | - | 1.38 | 40 |
| **VAS pain score (0 vs 4)** | 1.18 | 1.08 | - | 1.30 | 13 | 1.46 | 1.22 | - | 1.75 | 1 |
| **ASAT (U/L) (16 vs 42)** | 1.10 | 1.05 | - | 1.15 | 16 | 1.08 | 0.82 | - | 1.42 | 0 |
| **ALAT (U/L) (20 vs 43)** | 1.05 | 1.02 | - | 1.09 | 11 | 1.03 | 0.78 | - | 1.35 | 0 |
| **Alkaline phosphatase (U/L) (76 vs 152)** | 1.16 | 1.11 | - | 1.22 | 40 | 1.02 | 0.83 | - | 1.25 | 5 |
| **Bilirubine (µmol/L) (5 vs 13)** | 1.09 | 1.03 | - | 1.15 | 10 | 1.20 | 1.01 | - | 1.43 | 3 |
| **Gamma-GT (U/L) (29 vs 124)** | 1.14 | 1.10 | - | 1.18 | 61 | 1.21 | 0.97 | - | 1.51 | 16 |
| **Hb (mmol/L) ( 6 vs 8)** | 0.70 | 0.64 | - | 0.76 | 71 | 0.84 | 0.72 | - | 0.97 | 0 |
| **Thrombocytes (10^3/µL) (168 vs 337)** | 1.05 | 0.98 | - | 1.12 | 2 | 1.00 | 0.87 | - | 1.14 | 13 |
| **Leukocytes (10^9/L) (6 vs 12)** | 1.34 | 1.22 | - | 1.46 | 41 | 1.23 | 1.08 | - | 1.40 | 4 |
| **MCV (fL) (86 vs. 95)** | 0.95 | 0.89 | - | 1.02 | 2 | 1.03 | 0.93 | - | 1.14 | 0 |
| **RDW (fL) (13 vs 16)** | 1.29 | 1.17 | - | 1.41 | 28 | 1.05 | 0.87 | - | 1.26 | 2 |
| **LDH (U/L) (193 vs 305)** | 1.06 | 1.03 | - | 1.10 | 13 | 1.07 | 0.94 | - | 1.22 | 53 |
| **CRP (mg/L) (6 vs 76)** | 1.79 | 1.67 | - | 1.92 | 267 | 1.57 | 1.30 | - | 1.89 | 14 |
| **Glucose (mmol/l) (6 vs. 8)** | 1.26 | 1.19 | - | 1.34 | 65 | 1.21 | 1.07 | - | 1.36 | 8 |
| **Calcium (mmol/l) (2.3 vs 2.5)** | 0.85 | 0.79 | - | 0.91 | 23 | 0.95 | 0.87 | - | 1.04 | 14 |
| **Sodium (mmol/l) (135 vs 140)** | 0.62 | 0.57 | - | 0.67 | 137 | 0.85 | 0.76 | - | 0.95 | 14 |
| **Potassium (mmol/ml) (3.9 vs 4.5)** | 1.02 | 0.95 | - | 1.09 | 0 | 0.86 | 0.79 | - | 0.94 | 5 |
| **eGFR (ml/min) (58 vs 96)** | 0.74 | 0.68 | - | 0.80 | 58 | 0.57 | 0.35 | - | 0.93 | 18 |
| **Urea (mmol/L) (4.5 vs 8.5)** | 1.36 | 1.26 | - | 1.47 | 62 | 1.41 | 1.20 | - | 1.66 | 5 |
| **Creatinine (μmol/L) (66 vs 105)** | 1.09 | 1.05 | - | 1.13 | 23 | 0.63 | 0.39 | - | 1.02 | 9 |

# Supplemental Figure Legends

**Supplemental Figure 1.** Multivariable effects of the final predictors of hospitalization in the baseline model. The y-axis shows the logarithm of the odds, while the x-axis displays the predictor levels, with all other predictor effects set to their median values. Wald statistics are presented within each plot to indicate variable importance (higher values denote greater importance).

**Supplemental Figure 2.** Multivariable effects of the final predictors of hospitalization in the extended model. The y-axis shows the logarithm of the odds, while the x-axis displays the predictor levels, with all other predictor effects set to their median values. Wald statistics are presented within each plot to indicate variable importance (higher values denote greater importance).

**Supplemental Figure 3.** This figure shows the relationship between the hospitalization risk threshold and the sensitivity and specificity for the extended model.
