# Supplementary material for: Time to adjuvant chemotherapy and overall survival in advanced-stage ovarian cancer patients in England: a population-based retrospective cohort study
Source: ESMO Real World Data Digit Oncol. 2025 Apr 28;8:100143. doi: 10.1016/j.esmorw.2025.100143 (PMC12836497; doi:10.1016/j.esmorw.2025.100143)
Supplement: Supplementary Table 1 [file mmc6.docx]

| **Characteristic, *n*, %** | **Time to chemotherapy, days^1^** | ≤**6 weeks^1^** | **>6 weeks^1^** |
| --- | --- | --- | --- |
| Total cohort (N = 4,619) | 38 (30, 49) | 2,824 (61%) | 1,795 (39%) |
| **Age** |  |  |  |
| <60 years (N = 1,647) | 39 (30, 49) | 979 (59%) | 668 (41%) |
| 60-70 years (N = 1,659) | 37 (29, 47) | 1,054 (64%) | 605 (36%) |
| >70 years (N 1,313) | 39 (31, 49) | 791 (60%) | 522 (40%) |
| **Region** |  |  |  |
| East of England  (N = 658) | 36 (29, 45) | 442 (67%) | 216 (33%) |
| London (N = 570) | 35 (28, 44) | 410 (72%) | 160 (28%) |
| Midlands (N = 575) | 42 (34, 51) | 298 (52%) | 277 (48%) |
| North East & Yorkshire (N = 931) | 37 (28, 45) | 600 (66%) | 313 (34%) |
| North West (N = 631) | 43 (35, 55) | 298 (47%) | 333 (53%) |
| South East (N = 678) | 39 (31, 49) | 401 (59%) | 277 (41%) |
| South West (N = 594) | 37 (29, 47) | 375 (63%) | 219 (37%) |
| **Index of multiple deprivation** |  |  |  |
| 1 **-** most deprived (N = 649) | 41 (33, 50) | 358 (55%) | 291 (45%) |
| 2 (N = 825) | 39 (30, 49) | 484 (59%) | 341 (41%) |
| 3 (N = 992) | 38 (30, 48) | 629 (63%) | 363 (37%) |
| 4 (N = 1,074) | 38 (30, 48) | 672 (63%) | 402 (37%) |
| 5 - least deprived (N = 1,079) | 37 (29, 47) | 681 (63%) | 398 (37%) |
| **Ethnicity** |  |  |  |
| Asian (N = 185) | 37 (30, 48) | 120 (65%) | 65 (35%) |
| Black (N = 62) | 41 (35, 50) | 36 (58%) | 26 (42%) |
| Mixed Race N = (13) | 40 (34, 51) | 8 (62%) | 5 (38%) |
| Other (N = 65) | 38 (28, 44) | 46 (71%) | 19 (29%) |
| Unknown (N = 119) | 42 (34, 50) | 61 (51%) | 58 (49%) |
| White (N = 4,175) | 38 (30, 49) | 2,553 (61%) | 1,622 (39%) |
|  |  |  |  |

Supplementary Table 1: Comparison of median time to chemotherapy (days, IQR) by demographic factors of interest (age, region, Index of multiple deprivation, ethnicity).
