# Supplementary material for: Time to adjuvant chemotherapy and overall survival in advanced-stage ovarian cancer patients in England: a population-based retrospective cohort study
Source: ESMO Real World Data Digit Oncol. 2025 Apr 28;8:100143. doi: 10.1016/j.esmorw.2025.100143 (PMC12836497; doi:10.1016/j.esmorw.2025.100143)
Supplement: Supplementary Table 2 [file mmc7.docx]

| **Characteristic – primary surgery cohort** | **HR***^1^* | **95% CI***^1^* | **p-value** |
| --- | --- | --- | --- |
| **Time to chemotherapy** |  |  |  |
| ≤6 weeks (ref) | — | — |  |
| >6 weeks | 0.97 | 0.81, 1.15 | 0.7 |
| **Cancer stage** |  |  |  |
| 2B (ref) | — | — |  |
| 3 | 1.79 | 1.34, 2.40 | <0.001 |
| 4 | 2.58 | 1.76, 3.77 | <0.001 |
| **Age** |  |  |  |
| <60 (ref) | — | — |  |
| 60-70 | 1.16 | 0.94, 1.42 | 0.2 |
| 70< | 1.42 | 1.15, 1.76 | 0.001 |
| **Body mass index** |  |  |  |
| Underweight (<18.5) | 1.41 | 0.90, 2.21 | 0.14 |
| Normal weight (18.5-25, ref) | — | — |  |
| Overweight (25-30) | 0.82 | 0.67, 1.01 | 0.058 |
| Obese (30-40) | 0.86 | 0.68, 1.08 | 0.2 |
| Morbidly obese | 1.05 | 0.64, 1.74 | 0.8 |
| **Bevacizumab maintenance** | 1.24 | 0.99, 1.54 | 0.056 |
| **Region** |  |  |  |
| East of England (ref) | — | — |  |
| London | 0.99 | 0.68, 1.45 | >0.9 |
| Midlands | 0.96 | 0.66, 1.38 | 0.8 |
| North East & Yorkshire | 1.04 | 0.73, 1.46 | 0.8 |
| North West | 1.10 | 0.77, 1.57 | 0.6 |
| South East | 1.23 | 0.83, 1.82 | 0.3 |
| South West | 1.10 | 0.74, 1.64 | 0.6 |
| **Hospital type** |  |  |  |
| Teaching hospital (ref) | — | — |  |
| General hospital | 1.14 | 0.92, 1.41 | 0.2 |
| **Ethnicity** |  |  |  |
| White | — | — |  |
| Non-white | 1.07 | 0.80, 1.43 | 0.7 |
| **Index of multiple deprivation** |  |  |  |
| 1 - most deprived (ref) | — | — |  |
| 2 | 0.91 | 0.69, 1.20 | 0.5 |
| 3 | 0.65 | 0.49, 0.87 | 0.003 |
| 4 | 0.77 | 0.58, 1.02 | 0.070 |
| 5 - least deprived | 0.69 | 0.51, 0.93 | 0.015 |
| *^1^*HR = Hazard Ratio, CI = Confidence Interval | | | |

Supplementary Table 2: Complete-case analysis (excluding performance status as an explanatory variable for primary surgery cohort.
