# Supplementary material for: Time to adjuvant chemotherapy and overall survival in advanced-stage ovarian cancer patients in England: a population-based retrospective cohort study
Source: ESMO Real World Data Digit Oncol. 2025 Apr 28;8:100143. doi: 10.1016/j.esmorw.2025.100143 (PMC12836497; doi:10.1016/j.esmorw.2025.100143)
Supplement: Supplementary Table 3 [file mmc8.docx]

| **Characteristic – Interval surgery cohort** | **HR***^1^* | **95% CI***^1^* | **p-value** |
| --- | --- | --- | --- |
| **Time to chemotherapy** | — | — |  |
| ≤6 weeks (ref) | 1.21 | 1.06, 1.37 | 0.005 |
| >6 weeks |  |  |  |
| **Cancer stage** | — | — |  |
| 2B (ref) | 1.95 | 0.80, 4.75 | 0.14 |
| 3 | 2.16 | 0.88, 5.26 | 0.091 |
| 4 |  |  |  |
| **Age** | — | — |  |
| <60 (ref) | 1.25 | 1.09, 1.43 | 0.002 |
| 60-70 | 1.39 | 1.21, 1.61 | <0.001 |
| 70< |  |  |  |
| **Body mass index** | — | — |  |
| Underweight (<18.5) | 1.29 | 0.95, 1.76 | 0.10 |
| Normal weight (18.5-25, ref) | 0.92 | 0.81, 1.05 | 0.2 |
| Overweight (25-30) | 0.75 | 0.65, 0.87 | <0.001 |
| Obese (30-40) | 0.74 | 0.52, 1.03 | 0.078 |
| Morbidly obese | 1.11 | 0.98, 1.25 | 0.088 |
| **Bevacizumab maintenance** |  |  |  |
| **Region** | — | — |  |
| East of England (ref) | 0.71 | 0.57, 0.89 | 0.003 |
| London | 0.92 | 0.74, 1.15 | 0.5 |
| Midlands | 1.09 | 0.90, 1.32 | 0.4 |
| North East & Yorkshire | 0.87 | 0.71, 1.07 | 0.2 |
| North West | 0.84 | 0.68, 1.04 | 0.11 |
| South East | 0.91 | 0.73, 1.13 | 0.4 |
| South West |  |  |  |
| **Hospital type** | — | — |  |
| Teaching hospital (ref) | 0.94 | 0.82, 1.09 | 0.4 |
| General hospital |  |  |  |
| **Ethnicity** | — | — |  |
| White | 0.90 | 0.72, 1.12 | 0.3 |
| Non-white |  |  |  |
| **Index of multiple deprivation** | — | — |  |
| 1 - most deprived (ref) | 1.13 | 0.92, 1.39 | 0.3 |
| 2 | 0.92 | 0.75, 1.14 | 0.5 |
| 3 | 0.96 | 0.79, 1.17 | 0.7 |
| 4 | 0.92 | 0.75, 1.12 | 0.4 |
| *^1^*HR = Hazard Ratio, CI = Confidence Interval | | | |

Supplementary Table 3: Complete-case analysis (excluding performance status as an explanatory variable for interval surgery cohort.
